# Performance of ASA Polymer-Modified Asphalt Mixtures Under Aging Conditions

**DOI:** 10.3390/polym18131657

**Published:** 2026-07-03

**Authors:** Khalifa Salem Gallouz, Shaban Ismael Albrka Ali, Amina B. Abubakar, Faridah Hanim Khairuddin, Munder Bilema, Nasradeen Ali Khalifa, Mustafa Alas

**Affiliations:** 1 Department of Civil Engineering, College of Engineering, Misurata University, Misurata P.O. Box 2478, Libya; khalifa_g84@eng.misuratu.edu.ly; 2 Department of Civil and Construction Engineering, College of Engineering, A’Sharqiyah University, Ibra 400, Oman; 3 National Project for Disaster and Crisis Management, Libyan Authority for Scientific Research, Al-Nasser Street, Tripoli, Libya; 4 Department of Civil Engineering, Faculty of Engineering, Final International University, Via Mersin 10, 99320 Girne, North Cyprus, Turkey; amina.abubakar@final.edu.tr; 5 Department of Civil Engineering, Faculty of Engineering, Universiti Pertahanan Nasional Malaysia, Kem Sg Besi, Kuala Lumpur 57000, Malaysia; hanim@upnm.edu.my; 6 School of Civil Engineering, Tuanku Syed Sirajuddin Engineering Campus, Universiti Sains Malaysia, Nibong Tebal 14300, Penang, Malaysia; munderbilema@usm.my; 7 Faculty College of Engineering, Universiti Teknologi Mara, Shah Alam 40450, Selangor, Malaysia; nasradeen@uitm.edu.my; 8 Libyan Centre for Engineering Research and Information Technology, Bani Waleed 411, Libya; 9 Department of Civil Engineering, Faculty of Civil and Environmental Engineering, Near East University, Via Mersin 10, 99138 Nicosia, North Cyprus, Turkey; mustafa.alas@neu.edu.tr

**Keywords:** polymers, modified asphalt mixture, aging conditions, dynamic creep, wheel tracking, moisture susceptibility

## Abstract

The effects of weather conditions on modified asphalt mixtures were investigated in this study. Acrylonitrile Styrene Acrylate (ASA) polymer was used as a modifier with concentrations of 3, 5, and 7%. The viscosity test was performed to determine the blending and compaction temperatures for the base and modified mixtures, while Field Emission Scanning Electron Microscopy (FE-SEM) was utilized to explore the dispersion of the polymer in the asphalt binder matrix. Moreover, mechanical tests were applied to observe the changes in the modified asphalt binders. The highest improvements were obtained for a 5% ASA concentration. The resilient modulus increased by 78%, while resistance to dynamic creep improved by 74% compared with the base asphalt mixture. The wheel tracking and moisture susceptibility results further illustrated that the modified asphalt mixtures were less susceptible to moisture than the base asphalt mixture. The aging index results showed that the modifier can mitigate the effects of weather conditions, and the 5% ASA showed the best performance among the mixtures.

## 1. Introduction

In recent years, polymers have been regularly used as modifiers to modify asphalt binders [1,2] to mitigate several leading causes of asphalt mixtures distress that occur over the life of pavements [3,4,5,6]. Using polymers in the modification of asphalt binders and mixtures has the ability to enhance the quality of pavements, which results in safer road structures and to reduced maintenance costs [7,8]. However, using polymers in the modification of asphalt binders must not result in extreme hardness at low temperatures or high viscosity at elevated temperatures. The use of polymers to modify asphalt mixtures is a promising approach for improving their performance, which will increase the service lifetime of the pavements even though roads are subject to increasing traffic volumes [9,10,11,12]. Modification of asphalt mixtures using polymers demonstrated improved resistance to rutting and fatigue damage, temperature susceptibility, and stripping. Additionally, base asphalt binder is less viscous compared to modified asphalt binder. During the modification process, polymers blended with asphalt binders can enhance the resistance to asphalt distress at low temperatures without the modified asphalt becoming too brittle or too viscous at medium to high mixing temperatures. Iskender et al. [13], evaluated the stripping and rutting problems associated with asphalt mixtures by utilizing an SBS polymer and a fatty amine to modify asphalt mixtures. It was found that polymer-modified asphalt mixtures had reduced susceptibility to moisture compared to base asphalt mixtures. SBS-Kraton MD243, SBS-Kraton D1101, and EVAtane 2805 were used to modify asphalt binders (PG 58-34) and investigate the viscoelastic and mechanical properties of asphalt mixtures. The results of Marshall stability and flow values indicated that the SBS-modified asphalt mixtures had the maximum stability, while asphalt mixtures modified with EVA showed the lowest flow values. Furthermore, the asphalt mixtures modified with SBS were noted to have the highest elasticity, whereas those modified with EVA had the lowest values according to the stiffness index [14]. Moghaddam et al. [15] assessed the permanent deformation and the physical characteristics of base and modified asphalt mixtures with Polyethylene Terephthalate (PET) using the dynamic creep test at various temperatures and stress levels. The results obtained from their study showed that waste PET has a favorable influence on asphalt mixtures. The study that the permanent deformation resistance of samples modified with PET was enhanced in comparison with the base mixtures. Brovelli et al. [16], investigated the rutting resistance using two types of polymers, namely; low-density polyethylene (LDPE) and ethyl vinyl acetate (EVA). The concentrations of both additives were 3, 6, and 9% by weight of the asphalt binder. The rutting test results indicated that increasing the content of both polymers resulted in a substantial reduction in rutting depth. Furthermore, fatigue resistance was strongly improved.

Although polymer modification generally improves base binder properties, asphalt pavements remain highly susceptible to structural degradation throughout their service life due to the combined effects of continuous traffic loading and environmental exposure [17,18,19]. A primary cause of pavement distress is the progressive aging of the asphalt binder, a phenomenon that fundamentally alters the material’s rheological and chemical characteristics [20]. Aging typically manifests in two stages: volatilization of light components during construction (short-term aging) and continuous thermo-oxidative reactions in the field (long-term aging). Short-term aging alone has been shown to significantly impact the low-temperature cracking resistance of asphalt mixtures [21]. At the microstructural level, oxidative aging accelerates the conversion of aromatics to asphaltenes, modifying the nanoscale relaxation spectra and drastically increasing binder stiffness [22]. This chemical hardening process is further exacerbated by external environmental stressors, including prolonged exposure to ultraviolet radiation and moisture damage driven by precipitation or acid rain conditions [23,24].

To counter these complex degradation mechanisms, diverse polymer modifiers and plastic waste derivatives, such as polyethylene terephthalate (PET), have previously been incorporated to improve creep recovery, reduce temperature sensitivity, and enhance overall rutting performance [15,25,26]. The functional efficiency of these polymer-modified binders (PMBs) was strongly influenced by the polymer concentration and the integrity of the resulting microstructural network [27]. However, introducing polymeric compounds complicates the aging dynamics of the asphalt matrix. Studies evaluating both elastomeric and plastomeric modifiers reveal varying degrees of chemical shifts and rheological deterioration post-aging [28]. Styrene-butadiene-styrene (SBS), despite its widespread adoption, undergoes severe polymer chain scission under continuous oxidative stress [29,30]. The unsaturated butadiene segments within the SBS structure are inherently vulnerable to thermal and oxidative breakdown, necessitating the addition of antioxidants to delay degradation [31]. Consequently, mixtures modified with conventional polymers such as SBS and LDPE may experience significant performance deterioration when subjected to sever climatic conditions or combined aging and moisture conditioning [32,33]. The recognized vulnerabilities of traditional polymers have driven recent pavement engineering research toward the exploration of advanced alternative materials, including graphene-enhanced polymeric compounds and novel thermodynamic cooling materials, to preserve pavement durability [34,35].

Despite the development of various additives aimed at mitigating PMB degradation, there remains a critical need for binder modifiers possessing intrinsic chemical resistance to thermo-oxidative aging. Acrylonitrile Styrene Acrylate (ASA) presents a highly viable alternative. Distinct from conventional elastomers, ASA features a saturated acrylate rubber backbone that fundamentally resists the oxidative chain scission responsible for the failure of unsaturated polymers like SBS. While current literature extensively documents the aging mechanisms of standard PMBs, the rheological behavior and mechanical resilience of ASA-modified asphalt mixtures under prolonged aging conditions have not been systematically quantified. Therefore, this study aims to evaluate the performance of ASA polymer-modified asphalt mixtures under simulated unaged, short-term, and long-term aging conditions. By investigating the physical and morphological properties of the ASA-modified binder alongside the mechanical performance, focusing on permanent deformation and fatigue resistance of the resulting mixtures, this research seeks to determine the viability of ASA as a robust, age-resistant modifier for sustainable pavement infrastructure. Although various polymers have been widely investigated for asphalt mixture modification, limited studies have focused on the use of ASA as a modifier in asphalt mixtures. There is still insufficient understanding of how different ASA contents influence the performance characteristics of asphalt mixtures. Furthermore, previous studies have mainly concentrated on traditional polymers such as SBS and EVA, while the potential of ASA as an alternative modifier remains underexplored. Therefore, this study aims to evaluate the effect of incorporating 3, 5, and 7% ASA by weight of binder on the properties and performance of asphalt mixtures.

## 2. Materials and Testing Procedures

### 2.1. Materials

**Figure 1:**
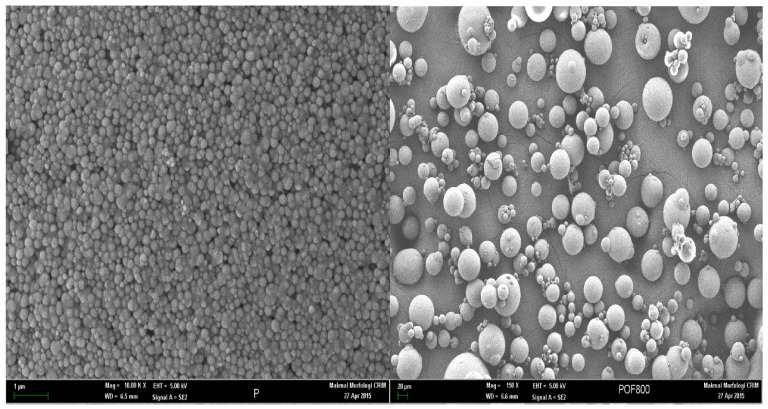
FE-SEM of Acrylate Styrene Acrylonitrile (ASA) polymer.

This investigation delineates the experimental procedure sequentially. The local Malaysian asphalt binder with penetration grade (60/70) and Acrylonitrile Styrene Acrylate (ASA) (Figure 1) were procured from a manufacture in China. The asphalt binder was modified incorporating ASA at concentrations of 3, 5, and 7% by the weight of asphalt binder (wt.%).

### 2.2. Modification Procedure of Asphalt Binders

The modification of base asphalt binders with ASA polymer was carried out in the laboratory under a high-shear mixer using the melting-blending method. The base asphalt binder was heated until it became liquid (150 °C), then mixed for 10 min before the temperature was raised to 170 ± 1 °C. Furthermore, the ASA polymer was added to the asphalt matrix and mixed at 5000 rpm, and the process was continued for 90 min to ensure adequate dispersion [36]. The homogeneity of the mixtures was assessed by performing a softening point test. Samples were collected at 30 min intervals, taken from the blend and evaluated until the softening point values reached relatively stable state.

### 2.3. Mixing Process of Modified HMA

Throughout the mixing process, the mixture (asphalt binders and aggregates) sufficient time must be allowed for the asphalt binder to be absorbed by aggregates and to ensure proper coating and adhesion resulting in a homogeneous asphalt mixture. For unmodified asphalt binders, the Asphalt Institute [37] recommends mixing temperatures corresponding to viscosity values of 0.17 ± 0.2 Pa·s and compaction temperatures corresponding to viscosity values of 0.28 ± 0.3 Pa·s. Meanwhile, for polymer-modified asphalt binders the appropriate mixing and compaction temperatures should correspond to viscosity ranging 0.28 and 0.55 Pa·s at a shear rate of 500 s^−1^ [4].

### 2.4. Aggregate Structure Design

**Figure 2:**
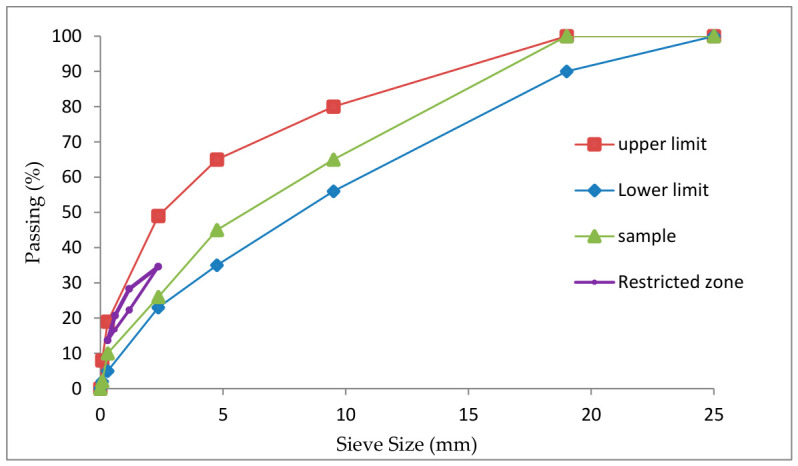
Aggregate Design of Asphalt Mixtures.

The method employed for structural design is the Superpave method with equivalent single axle loads (ESALs) less than 10^7^. Traffic levels is a key factor to define the requirements of mix design, including the aggregate physical properties, the number of gyrations required to compact the mixtures, and the volumetric properties. A nominal maximum aggregate size of 19.0 mm was selected for this research. Nevertheless, the selection of aggregates for designing asphalt mixtures is also dependent on cost, material type, local availability, and intended application of the pavement. The aggregate gradation used in the asphalt mix design in this investigation is shown in Figure 2.

### 2.5. Optimum Binder Content (OBC)

The mix design depends on an intermediate classification of traffic load level equal to or less than 10^7^ ESALs. Furthermore, the mix compatibility estimation was obtained at N_ini_ after 8 gyrations, and N_design_ of 96 gyrations was carefully selected to achieve the design density of the mix. Then, the compaction of samples was stopped after N_max_ (152) gyrations to avoid plastic failures initiated by traffic beyond the design levels. The properties of the mix were assessed for mixtures at the varying asphalt binder contents (4.5, 5, 5.5, 6, and 6.5% by the weight of the mix). The optimum asphalt binder content was selected using volumetric properties according to the Superpave mix design procedure. The asphalt mixtures were then compacted to provide a laboratory density equivalent to the estimated density under different traffic levels. To simulate expected traffic conditions, the number of gyrations was varied.

### 2.6. Superpave Mix Design Specimen Preparation

To regularize the influence of the asphalt binder, the samples required mixing and compaction at varying temperatures based on the rotational viscosities of the binders, ranging from 0.170 ± 20 to 0.280 ± 30 Pa·s. It should be noted that mixing temperatures beyond 175 °C might result in thermal degradation and therefore should not be used in road construction. Suppliers of asphalt binder should always refer to the recommendations for the appropriate laboratory and field temperatures (mixing and compaction) for modified asphalt binders. After the mixing process, loose test samples were subjected to conditioning, as specified in AASHTO PP-2 [38]. To regulate the volumetric characteristics of the mix design, the asphalt mixtures were placed in an oven for 120 min at the specified temperature required for compaction. During the curing process, loose mix samples were stirred every hour to ensure uniform aging for all samples. Moreover, the moulds were placed in an oven at 135 °C for at least 30–45 min before use. Sample compaction began at a pressure of 600 ± 18 kPa with a gyration angle of about 1.25° ± 0.02°. The volumetric properties of HMA must be controlled through the design and construction processes to ensure stable construction pavements. The Rice test was used to measure the theoretical maximum specific gravity (G_mm_) of asphalt mixtures along with the bulk specific gravity (G_mb_). The G_mm_ of loose mixtures and the G_mb_ of compacted mixtures were used to determine the air voids of the mix design. The bulk specific gravity (G_mb_) was calculated for all samples based on the ASTM D2726 [39], as expressed in Equation (1).

(1) $$G_{mb}=\frac{W_{D}}{W_{SSD}-W_{sub}}$$

where *W_D_* is the dry weight (g), and *W_SSD_* is the weight of the sample in saturated surface-dry condition (g), which is obtained by placing the sample in water for five minutes, then removing it from water, drying its surface by a clean towel, and measuring the mass. *W_Sub_* is the weight of the sample submerged in water (g).

Furthermore, the theoretical maximum specific gravity (G_mm_) of the asphalt mixtures was calculated based on the ASTM D2041 [40], as expressed in Equation (2).

(2) $$G_{mm}=\frac{P_{mm}}{\frac{p_{S}}{G_{se}}-\frac{P_{b}}{G_{b}}}$$

where *P_mm_* is the total loose mix and equals 100%, *P_s_* is the ratio of aggregate by the total weight of mix, *P_b_* is the asphalt ratio by total weight of mixtures, *G_se_* is the effective specific gravity of the given aggregates, whilst *G_b_* is the specific gravity of asphalt. Air voids in the compacted mix (*V_a_*) were calculated using the following Equation (3).

(3) $$V_{a}=\left(1-\frac{G_{mb}}{G_{mm}}\right)\times 100\;$$

where *G_mb_* is the bulk specific gravity of the asphalt mixtures, and *G_mm_* is the maximum specific gravity of the asphalt mixtures. Thus, volume of voids in mineral aggregates (*VMA*) was calculated using Equation (4).

(4) $$VMA=\left(1-\frac{G_{mb}(1-P_{b})}{G_{sb}}\right)\times 100\;$$

where *G_mb_* is the bulk specific gravity of the asphalt mix, *P_b_* is the asphalt percentage by total weight of mix, and G_sb_ is the bulk specific gravity of the given aggregate. Equation (5) below is used to compute the voids filled with asphalt (VFA):

(5) $$VFA=\left(\frac{VMA-V_{a}}{VMA}\right)\times \;100$$

## 3. Performance Tests of Asphalt Mixture

### 3.1. Indirect Tensile Resilient Modulus Test (M_R_)

The Universal Testing Machine (UTM-25) was employed to conduct the M_R_ tests. M_R_ is a basic parameter adopted to evaluate the mechanical and physical appearance of asphalt mixtures to provide a better mechanical and structural design for pavements. The M_R_ test is not considered a destructive test. It is expressed as the ratio of the applied stress to the recovered strain at a certain temperature and load. The sample had a diameter of 10 cm and a height of 6.35 ± 0.25 cm. Firstly, the samples were conditioned at the testing temperature in the machine chamber for 120 min. Then, the samples were subjected to five cycles of a haversine wave pattern followed by five loading pulses while the test was running, and the data were recorded. Moreover, the load was applied for a pulse duration of 100 ms, and the pulse repetition periods ranged from 300 to 1000 ms. Table 1 shows the M_R_ test parameters. For each mixture, temperature, and aging condition, three replicate specimens were tested (*n* = 3), and the average value was used for statistical comparison.

### 3.2. Dynamic Creep Test

This test was implemented for all mixtures using the procedure developed by Superpave Models (NCHRP 9-19) with a UTM machine. Three replicate samples with 4% air voids, a height of 63 ± 2 mm, and a diameter of 100 mm were prepared. The specimens were cured at 40 °C for four hours before the test started. The specifications of the procedure are tabulated in Table 2.

### 3.3. Wheel Tracking Test

This test represents a simulation of repeated movement of a wheel on the surface of a pavement. The technique used in this test was implemented at a laboratory scale using a Wessex wheel-tracking machine (Wessex Engineering and Metal Craft Co., Ltd., Frome, UK). The specimens were prepared for each mixture based on the designed aggregates gradation and OBC. In this study, 3700 g of aggregate were used to prepare samples with a height of 65 ± 1 mm and 150 mm in diameter, which were then placed in a slab mould with the aid of the Wessex wheel-tracking device. The mixture was compacted to satisfy the test requirement of 7 ± 0.5% air voids. The machine was adjusted to use the samples compacted by the Superpave Gyratory Compactor, and the air voids were determined by reducing the number of gyrations and validating the results using the Rice test. The rutting test was carried out at a specified temperature of 50 °C. The specimens were conditioned under dry test conditions for four hours prior to testing at the same temperature (50 °C). The test simulated traffic loading by applying 520 N of load for 45 min. Table 3 lists the test parameters, and an LVDT transducer was used to measure the rut deformation at the center of the slab. For the wheel tracking evaluation, three replicate specimens were prepared and tested for each mixture (*n* = 3).

### 3.4. Moisture Susceptibility Test

Moisture susceptibility is a critical parameter affecting the durability and performance of asphalt mixtures. It reflects the resistance of the binder-aggregate system to water-induced damage and loss of adhesion. In this study, it was evaluated using the AASHTO T283 procedure [41]. The cylindrical samples of base and modified asphalt mixtures with ASA were prepared with a diameter of 10 cm and height of 6.35 ± 0.25 cm compacted to achieve nearly 7 ± 1% air voids. For each mixture type, three specimens were tested under two conditions (wet and dry). The conditioned samples were subjected to partial vacuum saturation to achieve 70–80%, followed by a freezing stage and subsequent immersion in water at 60 °C for 24 h. All specimens were then tested using the indirect tensile strength (ITS) test. Moisture susceptibility was quantified using tensile strength ratio (TSR), defined as the ratio of the average ITS of conditioned samples to unconditioned samples. A minimum TSR value of 80% is commonly used as the acceptable criterion for satisfactory moisture resistance of asphalt mixture [42].

### 3.5. Aging Conditions

Pavement aging has a dominant effect on the asphalt binder’s stiffness and mixture performance due to oxidation and volatilization processes. In this study, aging was considered at two levels: short-term and long-term, and following the standard procedure. Short-term aging is caused by the loss of volatile materials and oxidation of asphalt during road construction in the field, whereas long-term aging was carried out in compliance with the AASHTO R30 method. The loose mixture was conditioned in an oven at a 135 ± 3 °C (or the selected compaction temperature, whichever is higher) for four hours, with manual stirring every 60 ± 5 min to ensure uniform exposure. After this process, the mixtures were compacted and prepared for performance testing, including dynamic creep and resilient modulus tests [43]. Long-term aging was simulated using the SHRP-A-383 procedures. Compacted specimens were placed in an oven at 85 °C for 120 h to represent extended filed aging (approximately 10 years of service life). After the conditioning, the samples were allowed to cool at room temperature (25–27 °C) for a minimum of 24 h before testing [43].

### 3.6. Statistical Analysis

All quantitative tests were evaluated using three replicate specimens for each mixture and condition (*n* = 3). Results are presented as mean ± standard deviation (SD), and the error bars represent SD. Statistical summaries and ANOVA results are reported immediately after the corresponding results. Statistical comparisons among ASA contents were conducted using one-way analysis of variance (ANOVA), followed by Tukey’s HSD post hoc test at a 95% confidence level (*p* < 0.05). Different lowercase letters above the bars and in the statistical tables indicate statistically significant differences among ASA contents within the same aging condition. Where 5% ASA and 7% ASA were statistically close, the optimum content was selected based on the overall balance of mechanical performance, aging resistance, moisture resistance, rutting resistance, and workability.

## 4. Results

### 4.1. Viscosity at 135 °C

**Figure 3:**
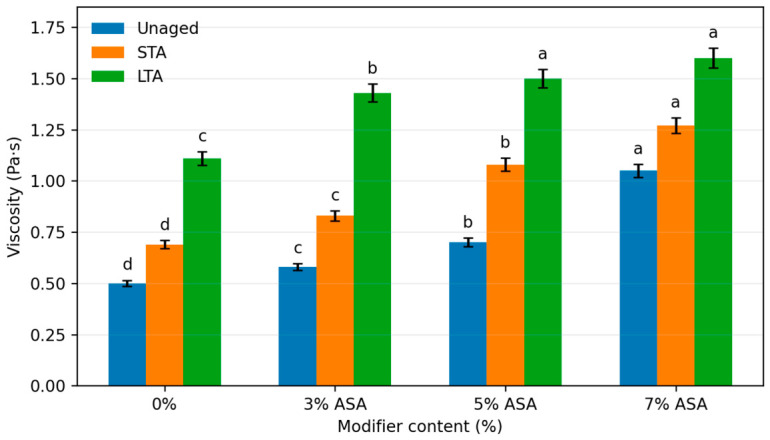
Viscosity Values before and after Aging. Viscosity values before and after aging. Bars show mean ± SD values from *n* = 3 replicates. Different lowercase letters indicate significant differences among ASA contents within the same aging condition according to Tukey’s HSD test (*p* < 0.05).

The viscosity of asphalt binder at elevated temperatures is considered one of the essential properties for selecting the working temperatures, as it represents the asphalt’s ability to pass through an asphalt plant. The viscosity of the base asphalt binder and ASA-modified asphalt binders is presented in Figure 3. The results indicate that the base asphalt binder has the lowest viscosity value compared with the asphalt binders modified with ASA polymer. Additionally, among the modified asphalt binders, it was observed that higher concentrations of modifiers produced higher viscosity values.

The statistical comparison confirmed that viscosity was significantly affected by ASA content under unaged, STA, and LTA conditions (ANOVA, *p* < 0.001 for all conditions). The 7% ASA binder showed the highest viscosity, whereas 5% ASA provided a substantial viscosity increase while remaining more workable than 7% ASA. Therefore, Figure 3 supports 5% ASA as the practical optimum when performance improvement and mixing/compaction workability are considered together.

The statistical summary Table 4 reports the mean viscosity values, standard deviations, coefficient of variation, and Tukey grouping for each ASA content under unaged, STA, and LTA conditions. The ANOVA Table 5 confirms that ASA content significantly affected viscosity under all aging conditions (*p* < 0.001). Although 7% ASA produced the highest viscosity, this is not necessarily beneficial because excessive viscosity may reduce workability during mixing and compaction. Therefore, the table supports the selection of 5% ASA as the practical optimum because it improves binder stiffness while avoiding the higher workability risk associated with 7% ASA.

Moreover, Table 6 presents the viscosity-aging index. The viscosity values increased under aging conditions, while the aging index decreased as the concentration of ASA polymer increased. According to SHRP conditions, the viscosity value must not be more than 3 Pa·s at 135 °C to ensure that the asphalt mixtures can be pumped. In general, all asphalt blends satisfied the SHRP specification requirements, regardless of the conditions.

### 4.2. Morphology of ASA Polymer

**Figure 4:**
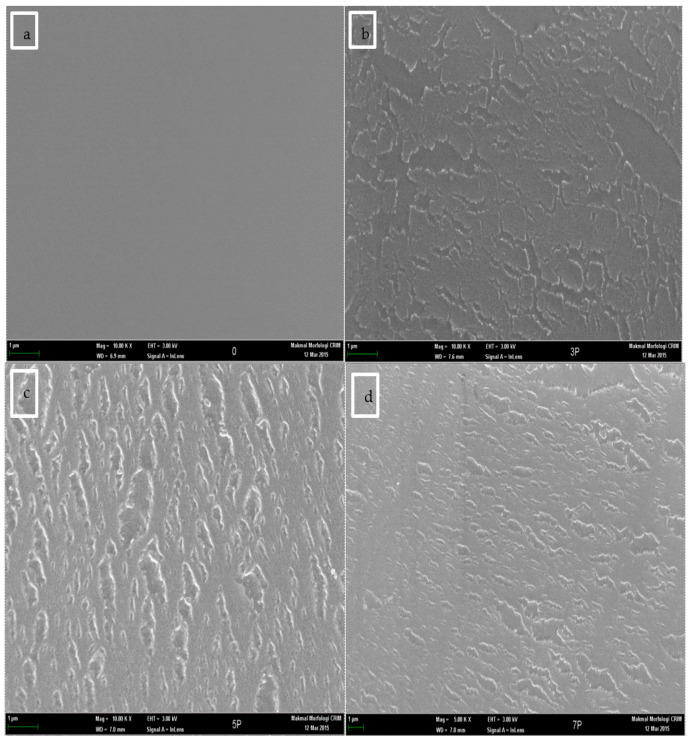
FE-SEM images of the Base and ASA Modified Asphalt binder, (**a**) base asphalt binder, (**b**) 3% ASA, (**c**) 5%ASA and (**d**) 7%ASA.

The compatibility between polymers and asphalt binder is a critical issue for polymer-modified asphalt binder [44]. Therefore, the morphology of ASA-modified asphalt binder was evaluated using Field Emission Scanning Electron Microscopy (FE-SEM) to determine the fineness and distribution of the polymer within the asphalt binder matrix. Figure 4 shows that an increase in ASA polymer concentration has a remarkable effect on the compatibility of modified asphalt binders, as the storage stability results indicate that ASA polymer can be stably incorporated up to 5%. It can be observed from Figure 4 that the wrinkled surfaces depict the entanglement between ASA polymer particles and asphalt matrix, while the particles are also uniformly distributed, which indicates that the asphalt binder may exhibit good performance. A uniform and regular distribution within a continuous asphalt phase helps to prevent agglomeration among polymer particles and phase separation between the polymer and bitumen.

### 4.3. Resilient Modulus Test

**Figure 5:**
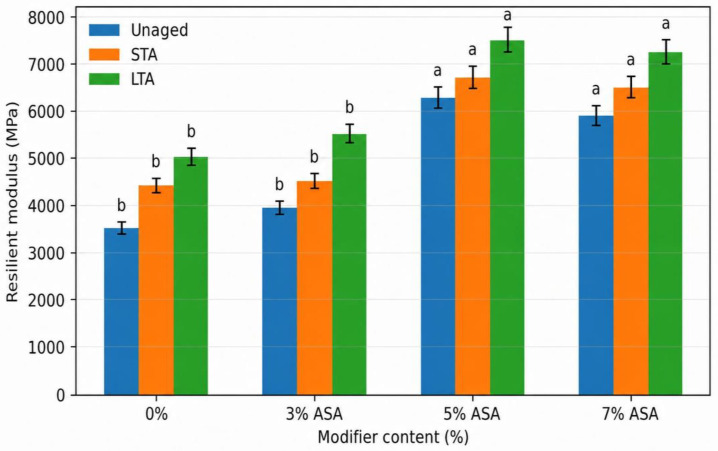
Resilient Modulus of the Modified and Base Asphalt Mixtures at 25 °C.

**Figure 6:**
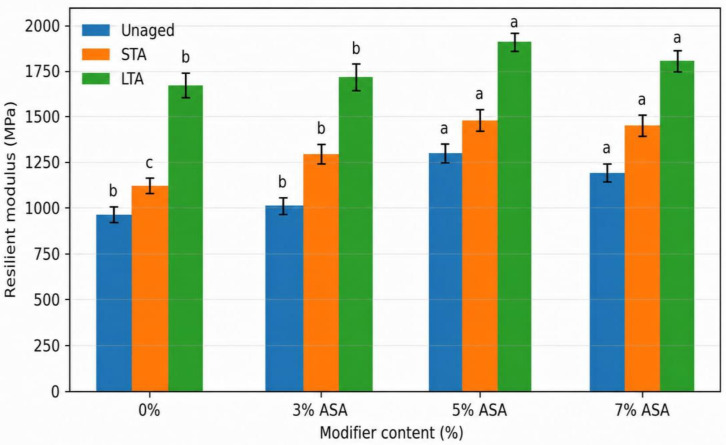
Modulus of Resilience for the Modified and Base Asphalt Mixtures at 40 °C.

Permanent pavement deformation has a significant impact on the performance and service life of asphalt pavements. Permanent deformation does not simply reduce the service life of the pavement but can also affect vehicle handle, which can be risky for road users [45]. The factors affecting the depth and rate of permanent deformation are the traffic volume and loading, tire pressure, temperature, aggregate and mix properties, the thickness of asphalt mixtures, and the type of asphalt binder. Additionally, the main contributing factors are high temperatures and traffic loads [46]. The blend of the materials (asphalt, aggregates, and polymer) results in improvements in the rutting resistance. Moreover, the increasing the ASA polymer content resulted in a reduction in the deformation at elevated temperatures (50 °C). As shown in Figure 5 and Figure 6, it was observed that 5% ASA produced the best resistance in terms of the resistance rutting performance, whereas the base asphalt mixtures showed the lowest performance among the mixtures. The use of ASA polymer to modify the base asphalt mixtures is significantly more effective than the use of the base asphalt mixture alone. The improvement in rutting resistance of the ASA-modified mixtures is attributed to the modification of viscoelastic behavior of the binder at high temperatures. It is probable that the elastic recovery and stiffness of the binder system were improved with the addition of ASA polymer, which reduced the tendency of asphalt mixtures to undergo viscous flow and permanent shear deformation under repeated loading. The 5% ASA shows better performance, which indicates that this modifier concentration has reached the best balance between stiffness enhancement and structural stability in the asphalt matrix. However, high polymer content can negatively affect the homogeneity and compatibility of the binders, which may limit the effectiveness of stress distribution in the mixture. Furthermore, the reduction in permanent deformation at 50 °C indicates that the ASA modification reduced the temperature susceptibility of asphalt binder, enabling the mixture to maintain its load-bearing capacity and structural integrity under severe thermal and traffic loading conditions. These results are consistent with those reported in previous studies in which SBS and CRM were used to modify asphalt mixtures [47].

Bars show mean ± SD values from *n* = 3 replicates (Figure 5). Different lowercase letters indicate significant differences among ASA contents within the same aging condition according to Tukey’s HSD test (*p* < 0.05).

At 25 °C, ASA content significantly affected the resilient modulus under all aging conditions (ANOVA, *p* < 0.001). The 5% ASA mixture achieved the highest mean modulus in the unaged, STA, and LTA conditions. Although 7% ASA was statistically close to 5% ASA in some cases, its mean values were lower, indicating that increasing ASA beyond 5% did not provide additional stiffness benefit.

The statistical summary Table 7 shows the resilient modulus at 25 °C for each ASA content and aging condition. The mean ± SD values provide the basis for the error bars, while the Tukey letters identify statistical groupings among mixtures. The ANOVA Table 8 shows significant differences among ASA contents (*p* < 0.001). The 5% ASA mixture had the highest resilient modulus in the unaged, STA, and LTA conditions. Although 7% ASA was statistically close to 5% ASA in some cases, 5% ASA provides the better overall choice because it achieves the highest stiffness response with lower viscosity than 7% ASA.

In Figure 6, bars show mean ± SD values from *n* = 3 replicates. Different lowercase letters indicate significant differences among ASA contents within the same aging condition according to Tukey’s HSD test (*p* < 0.05).

At 40 °C, the effect of ASA content on resilient modulus was statistically significant for unaged (*p* < 0.001), STA (*p* < 0.001), and LTA (*p* = 0.0095) mixtures. The 5% ASA mixture produced the highest mean modulus under all aging conditions, while 7% ASA remained close but lower. This result further supports 5% ASA as the optimum content at elevated temperature.

The statistical summary Table 9 presents the resilient modulus at 40 °C, which is important for evaluating mixture performance at elevated temperature. The ANOVA Table 10 shows results indicate that ASA content significantly influenced the resilient modulus under unaged, STA, and LTA conditions. The 5% ASA mixture generally produced the highest modulus, while 7% ASA remained close but did not provide a consistent additional benefit. This supports the conclusion that increasing ASA beyond 5% does not necessarily improve high-temperature stiffness and may compromise workability.

These effects indicate that using ASA polymer as a modifier produces asphalt mixtures with higher toughness and load-bearing capacity. As a result, the resilient modulus was evaluated under two aging conditions: short-term (STA) and long-term (LTA). The aging index was calculated to assess the impact of aging on the modified asphalt mixtures. For all mixtures, regardless of temperature, the aging index value is expected to increase with aging. Due to the influence of temperature on the softening point of the asphalt binder, the aging index is highly temperature-dependent. Table 11 and Table 12 present the aging index of the modified asphalt mixtures. It was observed that increasing the modifier content reduced the aging index, indicating that the ASA polymer has the ability to resist and delay aging effects. Moreover, the mixture containing 5% ASA showed the lowest aging response among all the asphalt mixtures.

### 4.4. Dynamic Creep

**Figure 7:**
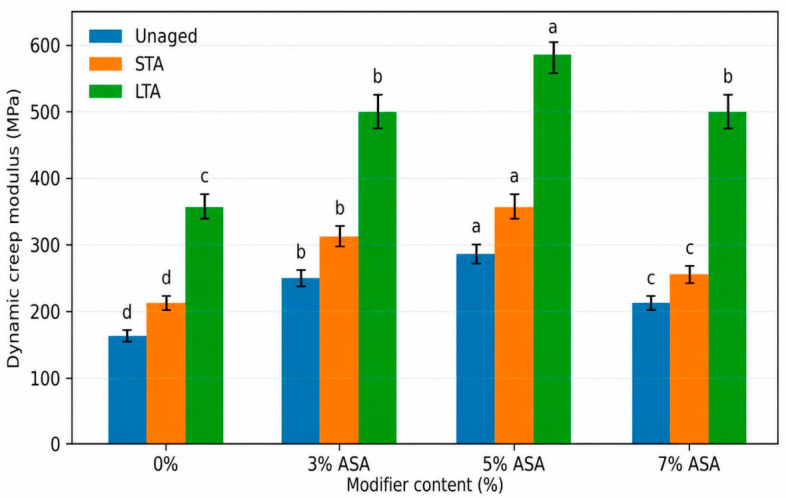
Dynamic Creep Modulus of the Modified and Base Asphalt Mixtures at 40 °C.

**Figure 8:**
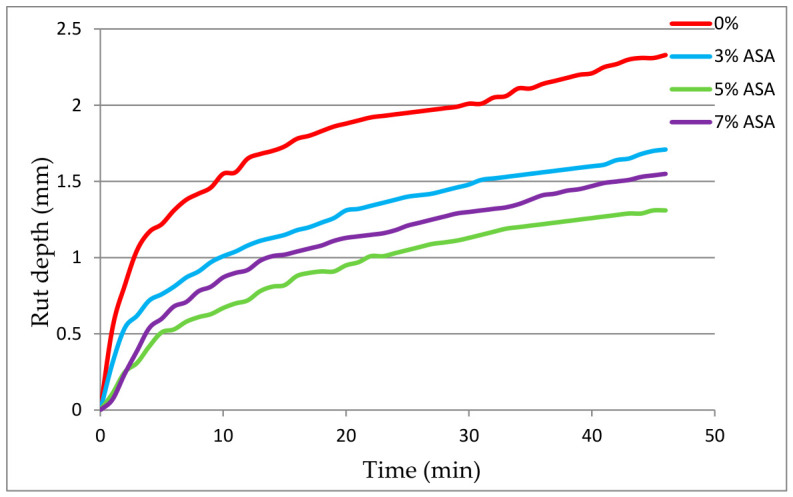
Wheel Tracking of the Modified and Base Asphalt Mixtures.

Applying a load to the surface of asphalt mixtures causes deformation; however, most of the deformation is recovered once the load is removed due to the viscoelastic properties of the material. Nonetheless, a small amount of non-recoverable viscous deformation remains within the mixtures. Moreover, when multiple load cycles are applied, the accumulation of these minor deformations results in permanent deformation on the surface of the asphalt mixtures. The effects of the dynamic creep modulus of the modified and base asphalt mixture are presented in Figure 7. It was found that with an increase in the modifier concentration, the stiffness of the mixtures improved up to 5% ASA. The mixture with 5% ASA showed better resistance to rutting (higher stiffness) among the mixtures, whereas the base asphalt mixtures were observed to have the lowest resistance to deformation (lower stiffness). The mixture containing 7% ASA showed a decrease in stiffness compared with 5% ASA, which may be due to the phase segregation between the asphalt and ASA polymer, leading to weakness in the asphalt mixture. This test was also used to evaluate the STA and LTA aging conditions of the base and modified asphalt mixtures. Figure 8 illustrates that the irreversible deformation of the modified asphalt mixtures decreased as the stiffness of the asphalt mixture increased after STA and LTA. In other words, temperature dependence of permanent deformation in modified mixture samples was considerably reduced compared with the base asphalt samples. The observed improvement in dynamic creep performance indicates that the ASA modification shifted the viscoelastic balance of the asphalt binder toward a more elastic response under cyclic loading. This behavior restricts the flow of binder and enhances the strain recovery after load removal, thus decreasing the buildup of plastic strain. The better performance at 5% ASA indicates that this dosage had formed a more robust polymer–binder network that could enhance stress transfer within the asphalt matrix. However, the reduction in stiffness at 7% ASA could be an indication of reduced compatibility and non-uniform dispersion of polymers, which may undermine the continuity of the binder phase and adversely affect the efficiency of load distribution. Furthermore, the reduced sensitivity of the modified mixtures to the STA and LTA aging indicated that the modification of ASA improved the thermo-oxidative stability of the binder, and thus, maintained the mechanical properties of the asphalt mixture under long-term aging and high-temperature conditions.

Bars in Figure 7, show mean ± SD values from *n* = 3 replicates. Different lowercase letters indicate significant differences among ASA contents within the same aging condition according to Tukey’s HSD test (*p* < 0.05).

Dynamic creep modulus differed significantly among ASA contents for all aging conditions (ANOVA, *p* < 0.001). The 5% ASA mixture gave the highest creep modulus in the unaged, STA, and LTA states, indicating the best resistance to accumulated permanent deformation. The lower response of 7% ASA suggests that excessive polymer content did not further improve the polymer-binder network.

The statistical summary Table 13 and ANOVA Table 14 demonstrate that dynamic creep modulus was significantly affected by ASA content for all aging conditions (*p* < 0.001). The 5% ASA mixture achieved the highest dynamic creep modulus, indicating the strongest resistance to permanent deformation. The 7% ASA mixture improved performance compared with the base mixture, but its lower values compared with 5% ASA suggest that the optimum polymer-binder network was achieved at 5% ASA.

Table 15 compares the changes in the base and ASA polymer-modified samples after STA and LTA using the aging index calculated from the dynamic creep test. When the modifier content increased to 5%, the aging index decreased slightly. This behavior is attributed to the ability of the polymer to delay aging. Higher resistance to aging was observed when the modifier concentration was 5%, whereas the base asphalt mixtures showed higher aging index values under STA and LTA conditions. However, when the polymer content in asphalt mixture exceeded 5%, phase separation occurred between the ASA polymer and asphalt binder. This phenomenon led to increased temperature susceptibility and reduced resistance to rutting and aging at elevated temperatures.

### 4.5. Wheel Tracking Test Results

Permanent pavement deformation has a dominant effect on the performance and lifecycle of asphalt pavements. Irreversible deformation does not only reduce the service life of the pavement but could also affect vehicle handling, which can pose risks to road users [45]. The factors affecting the depth and rate of permanent deformation include traffic loading and volume, tire pressure, temperature, aggregate and mixture properties, the thickness of asphalt mixtures, and the type of asphalt binder. Additionally, high temperatures and traffic loads are considered the main contributing factors [46]. The combination of materials (asphalt, aggregates, and polymer) results in improved resistance to rutting. Moreover, increasing the ASA polymer content reduced permanent deformation at elevated temperatures (50 °C). From Figure 8, it was observed that the mixture with 5% of the modifier exhibited the highest resistance to rutting performance, whereas the base asphalt mixtures showed the lowest performance among the mixtures. The use of ASA polymer to modify the base asphalt mixture was found to be significantly more effective than the use of base asphalt mixtures alone. Mechanistically, the enhanced viscoelastic response of the binder phase and the consequent reduction in time-dependent strain accumulation under sustained loading can explain the enhanced rutting resistance of ASA-modified asphalt mixtures. The addition of polymeric modifiers changes the rheological balance of the asphalt system by increasing the elastic component and reducing the viscous flow, which is the main cause of permanent deformation at high service temperatures. Such transformation results in better stress redistribution in the mastic and fewer local shear concentrations under repeated wheel loading. Furthermore, the ASA polymer is expected to enhance the internal microstructure of asphalt mastic due to the increased polymer-bitumen interaction and the formation of a more stable three-dimensional network. Such a structure improves the load transfer efficiency between aggregate particles and results in improved aggregate interlock and less particle reorientation under traffic loading. However, high polymer dosage may influence phase compatibility and induce heterogeneity of the binder phase, which could deteriorate the structural continuity and reduce the rutting resistance. Additionally, the reduction in temperature susceptibility demonstrates the enhanced thermo-mechanical stability of the modified mixtures where these mixtures can preserve their stiffness and structural integrity at higher thermal conditions. This behaviour is particularly important in hot climates where asphalt binders are susceptible to softening and rapid deformation. The results generally show that polymer modification improves the macroscopic rutting performance and improves the internal load bearing mechanisms that control the permanent deformation resistance of asphalt mixtures fundamentally. These outcomes are consistent with other findings from other studies in which SBS and CRM were used to modify the asphalt mixtures [47].

The dynamic stability was calculated from wheel tracking test results. It is expressed as the number of wheel passes per 1 of rut depth during the last 15 min of a wheel-tracking test lasting 1 h. The dynamic stability was calculated according to Japanese standards using Equation (6).

(6) $$Ds=\frac{360}{D_{2520}-D_{1890}}$$

where:

Ds = the dynamic stability (pass/mm);
D_2520_ = deformation at 2520 passes;
D_1890_ = deformation at 2520 passes.

**Figure 9:**
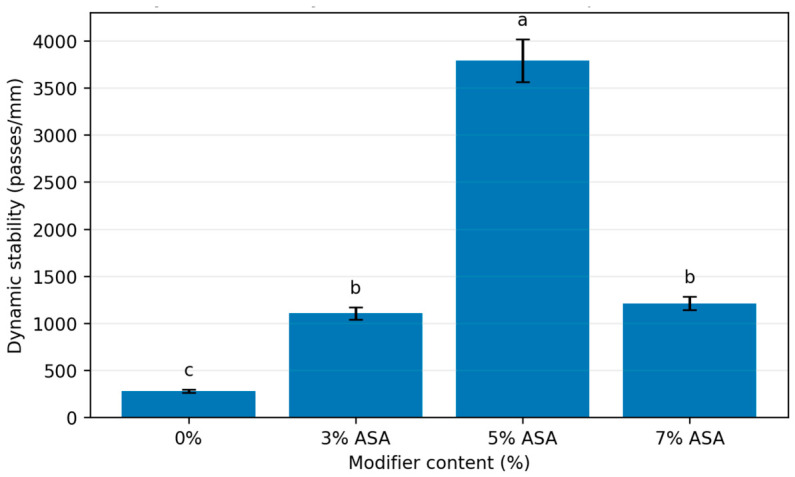
Dynamic stability of the Modified and Base Asphalt Mixtures.

The dynamic stability of the base and ASA polymer-modified asphalt mixtures is illustrated in Figure 9. It was observed that the base asphalt samples had the lowest dynamic stability compared with the modified asphalt mixtures. Moreover, the mixture with 5% ASA showed the highest dynamic stability among the modified asphalt mixtures, indicating that it was greater resistance to permanent deformation. Dynamic stability increased with increasing modifier content up to a concentration of 5%. A dynamic stability value greater than 3000 passes/mm is generally recommended for heavily traffic roads. For the mixture with 5% ASA, the dynamic stability improved by up to 358%.

Bars in Figure 9, show mean ± SD values from *n* = 3 replicates. Different lowercase letters indicate significant differences among ASA contents according to Tukey’s HSD test (*p* < 0.05).

The dynamic stability results showed a statistically significant difference among mixtures (ANOVA, *p* < 0.001). The 5% ASA mixture exhibited the highest dynamic stability and was significantly higher than the base, 3% ASA, and 7% ASA mixtures, confirming that 5% ASA provided the strongest rutting resistance in the wheel tracking evaluation.

The statistical summary Table 16 shows that 5% ASA produced the highest dynamic stability, while the ANOVA Table 17 confirms a statistically significant difference among mixtures (*p* < 0.001). Because dynamic stability is directly related to rutting resistance, this table provides strong support for selecting 5% ASA as the optimum modifier content. The lower dynamic stability of 7% ASA indicates that adding more ASA did not further improve rutting resistance.

### 4.6. Moisture Susceptibility Test Results

**Figure 10:**
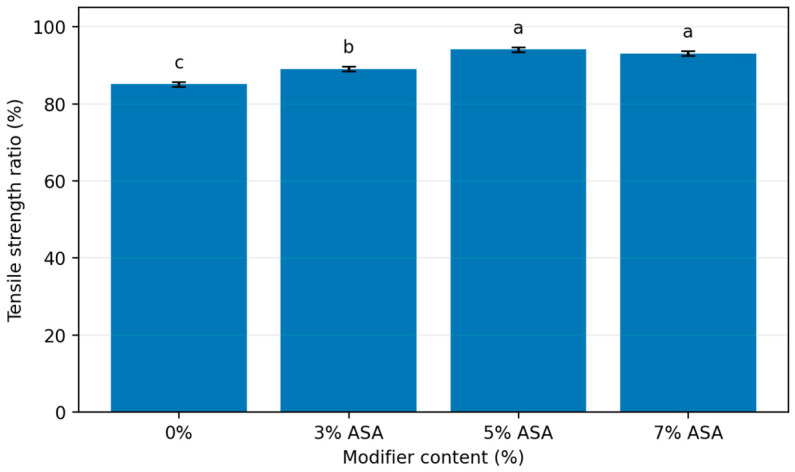
Moisture Susceptibility of the Modified and Base Asphalt Mixtures.

Stripping is characterized by the loss of adhesion between the asphalt binder and the aggregate surface. While the exact mechanisms driving this phenomenon are complex and not yet fully understood [48], stripping progressively reduces the pavement’s service life. This degradation ultimately manifests as various surface distresses, including raveling, cracking, corrugation, rutting, and shoving [49]. To evaluate moisture susceptibility, the Tensile Strength Ratio (TSR) test was conducted in accordance with the AASHTO T283 standard. This test was used to assess whether polymer modification inadvertently increases moisture vulnerability and determines the efficacy of the ASA polymer as a potential anti-stripping agent [13]. As depicted in Figure 10, all evaluated asphalt mixture samples—regardless of the modifier concentration—achieved a TSR value exceeding the 80% minimum threshold specified by the AASHTO T283 guidelines [42]. The base asphalt mixture exhibited a TSR of 85%. The introduction of the ASA polymer significantly enhanced the adhesion between the binder and the aggregates. The modifier effectively coats the aggregate particles, forming a protective barrier that improves overall resistance to moisture-induced damage. Specifically, increasing the ASA polymer content up to 5% exhibited the highest TSR value moisture resistance, mixture containing 5% ASA demonstrating an 11% improvement in TSR compared to the base mix. However, at a higher concentration of 7% ASA, improvement slightly diminished to 9%. These findings align with previous studies on Styrene-Butadiene-Styrene (SBS) and Epoxidized Natural Rubber (ENR) modifiers, which have also reported to reduce the moisture susceptibility of asphalt mixtures. Overall, the polymer-modified mixtures exhibited higher TSR values and enhanced durability compared with the unmodified base asphalt mixture [50].

In Figure 10, bars show mean ± SD values from *n* = 3 replicates. Different lowercase letters indicate significant differences among ASA contents according to Tukey’s HSD test (*p* < 0.05).

The TSR values differed significantly among mixtures (ANOVA, *p* < 0.001). All ASA-modified mixtures exceeded the 80% TSR acceptance criterion, demonstrating satisfactory moisture resistance. Although 5% ASA and 7% ASA were statistically close, 5% ASA recorded the highest mean TSR while maintaining lower viscosity than 7% ASA; therefore, it offered the best balance between moisture resistance and workability.

Statistical summary Table 18 presents TSR values for moisture susceptibility, and the ANOVA Table 19 confirms significant differences among mixtures (*p* < 0.001). All ASA-modified mixtures exceeded the 80% TSR acceptance criterion, indicating satisfactory moisture resistance. The 5% ASA mixture produced the highest TSR, while 7% ASA was very close and shared the same Tukey letter, indicating that they may not be statistically different. Therefore, 5% ASA is justified as the optimum because it provides comparable or better moisture resistance while maintaining lower viscosity and better overall workability than 7% ASA.

## 5. Conclusions

This study investigated the behavior of asphalt mixtures modified with ASA polymer under unaged, short-term aged, and long-term aged conditions, using mechanical performance tests using different polymer contents (0, 3, 5, and 7 wt.%). The main findings are summarized as follows: Overall, the statistical analysis confirms that 5% ASA is the optimum modifier content because it consistently provided the best or statistically comparable performance across resilient modulus, dynamic creep, dynamic stability, and moisture susceptibility, while avoiding the higher viscosity and potential workability limitations associated with 7% ASA.

- The resilience modulus of ASA-modified asphalt mixtures was higher than that the base asphalt mixture. The mixture containing 5% ASA consistently showed the best performance among all mixtures. Increasing the modifier content improved the rheological properties of the modified asphalt mixtures. This improvement may be attributed to higher viscosity, which leads to enhanced resilience properties. These results indicate that the use of modified asphalt binders produces asphalt mixtures capable of withstanding daily traffic loads.
- The dynamic creep results indicated a clear improvement in rutting resistance with the addition of ASA. This enhancement is attributed to better interaction between ASA polymer and asphalt binder, leading to a more stable internal structure. Although permanent deformation increased with aging due to increased stiffness, aged modified mixtures still performed better than the unmodified mixture. In addition, the aging index of modified mixtures was lower than that of the control, indicating improved resistance to aging.
- Rutting performance further confirmed the beneficial effect of ASA modification. The modified mixtures exhibited improved resistance to permanent deformation, with optimum performance observed up to 5% ASA content.
- The TSR results showed that ASA-modified mixtures exhibited improved moisture resistance compared to the control mixture, whereas the base asphalt mixture was more susceptible to moisture damage.

## Figures and Tables

**Table 1:** Test Parameters for Resilient Modulus.

Parameter	Condition
Temperature (°C)	25 and 40
Loading pulse width (ms)	100
Pulse repetition period (ms)	1000
Applied load at 25 °C	10% of the indirect tensile strength (500 N)
Applied load at 40 °C	10% of the indirect tensile strength (300 N)

**Table 2:** Dynamic creep test parameters.

Parameter	Condition
Temperature (°C)	40
Loading pulse width (ms)	100
Applied load (KPa)	100
Termination	After 10,000 με or 3600 Cycles

**Table 3:** Wheel Trucking Test parameters.

Parameter	Condition
Temperature (°C)	50
Loading pulse width (ms)	21
Applied load (N)	520
Termination	>45 min or depth > 15 mm

**Table 4:** Statistical summary for Viscosity Values before and after Aging.

Condition	ASA Content	*n*	Mean ± SD	CV (%)	Tukey Letter
Unaged	0%	3	0.500 ± 0.015	3.0	d
Unaged	3% ASA	3	0.580 ± 0.017	3.0	c
Unaged	5% ASA	3	0.700 ± 0.021	3.0	b
Unaged	7% ASA	3	1.050 ± 0.031	3.0	a
STA	0%	3	0.690 ± 0.021	3.0	d
STA	3% ASA	3	0.830 ± 0.025	3.0	c
STA	5% ASA	3	1.080 ± 0.032	3.0	b
STA	7% ASA	3	1.270 ± 0.038	3.0	a
LTA	0%	3	1.110 ± 0.033	3.0	c
LTA	3% ASA	3	1.430 ± 0.043	3.0	b
LTA	5% ASA	3	1.500 ± 0.045	3.0	a
LTA	7% ASA	3	1.600 ± 0.048	3.0	a

**Table 5:** ANOVA results for Viscosity Values before and after Aging.

Condition	F-Value	*p*-Value
Unaged	360.376	<0.001
STA	225.445	<0.001
LTA	73.973	<0.001

**Table 6:** Aging Index of Viscosity before and After Aging.

Asphalt	Unaged	RTFOT	PAV	Aging Index	
				RTFOT/Unaged	PAV/Unaged
Base asphalt	0.5	0.69	1.11	1.38	2.22
3% ASA	0.58	0.83	1.43	1.43	2.47
5% ASA	0.7	1.08	1.5	1.54	2.14
7% ASA	1.05	1.27	1.6	1.2	1.52

**Table 7:** Statistical summary for Resilient Modulus of the Modified and Base Asphalt Mixtures.

Condition	ASA Content	*n*	Mean ± SD	CV (%)	Tukey Letter
Unaged	0%	3	3528.00 ± 123.48	3.5	b
Unaged	3% ASA	3	3953.00 ± 138.36	3.5	b
Unaged	5% ASA	3	6293.00 ± 220.26	3.5	a
Unaged	7% ASA	3	5906.00 ± 206.71	3.5	a
STA	0%	3	4422.00 ± 154.77	3.5	b
STA	3% ASA	3	4524.00 ± 158.34	3.5	b
STA	5% ASA	3	6713.00 ± 234.95	3.5	a
STA	7% ASA	3	6507.00 ± 227.74	3.5	a
LTA	0%	3	5037.00 ± 176.30	3.5	b
LTA	3% ASA	3	5529.00 ± 193.52	3.5	b
LTA	5% ASA	3	7523.00 ± 263.31	3.5	a
LTA	7% ASA	3	7260.00 ± 254.10	3.5	a

**Table 8:** ANOVA results for Resilient Modulus of the Modified and Base Asphalt Mixtures.

Condition	F-Value	*p*-Value
Unaged	182.442	<0.001
STA	117.701	<0.001
LTA	90.926	<0.001

**Table 9:** Statistical summary for Modulus of Resilience for the Modified and Base Asphalt Mixtures.

Condition	ASA Content	*n*	Mean ± SD	CV (%)	Tukey Letter
Unaged	0%	3	965.00 ± 38.60	4.0	b
Unaged	3% ASA	3	1014.00 ± 40.56	4.0	b
Unaged	5% ASA	3	1299.00 ± 51.96	4.0	a
Unaged	7% ASA	3	1194.00 ± 47.76	4.0	a
STA	0%	3	1118.00 ± 44.72	4.0	c
STA	3% ASA	3	1296.00 ± 51.84	4.0	b
STA	5% ASA	3	1482.00 ± 59.28	4.0	a
STA	7% ASA	3	1452.00 ± 58.08	4.0	a
LTA	0%	3	1677.00 ± 67.08	4.0	b
LTA	3% ASA	3	1730.00 ± 69.20	4.0	b
LTA	5% ASA	3	1940.00 ± 77.60	4.0	a
LTA	7% ASA	3	1820.00 ± 72.80	4.0	a

**Table 10:** ANOVA results for Modulus of Resilience for the Modified and Base Asphalt Mixtures.

Condition	F-Value	*p*-Value
Unaged	35.861	<0.001
STA	28.992	<0.001
LTA	7.716	0.0095

**Table 11:** Aging Index of Modified Asphalt Mixtures at 25 °C.

Asphalt Mixture	Unaged	STA	LTA	Aging Index	
				STA/Unaged	LTA/Unaged
Base mixtures	3528	4422	5037	1.25	1.43
3% ASA	3953	4524	5529	1.14	1.40
5% ASA	6293	6713	7523	1.07	1.20
7% ASA	5906	6507	7260	1.10	1.23

**Table 12:** Aging Index of Modified Asphalt Mixtures at 40 °C.

Asphalt Mixture	Unaged	STA	LTA	Aging Index	
				RTFOT/Unaged	PAV/Unaged
Base mixtures	965	1118	1677	1.16	1.74
3% ASA	1014	1296	1730	1.29	1.71
5% ASA	1299	1482	1940	1.14	1.49
7% ASA	1194	1452	1820	1.22	1.52

**Table 13:** Statistical summary for Dynamic Creep Modulus of the Modified and Base Asphalt Mixtures.

Condition	ASA Content	*n*	Mean ± SD	CV (%)	Tukey Letter
Unaged	0%	3	164.00 ± 8.20	5.0	d
Unaged	3% ASA	3	250.00 ± 12.50	5.0	b
Unaged	5% ASA	3	286.00 ± 14.30	5.0	a
Unaged	7% ASA	3	213.00 ± 10.65	5.0	c
STA	0%	3	213.00 ± 10.65	5.0	d
STA	3% ASA	3	313.00 ± 15.65	5.0	b
STA	5% ASA	3	357.00 ± 17.85	5.0	a
STA	7% ASA	3	256.00 ± 12.80	5.0	c
LTA	0%	3	357.00 ± 17.85	5.0	c
LTA	3% ASA	3	500.00 ± 25.00	5.0	b
LTA	5% ASA	3	588.00 ± 29.40	5.0	a
LTA	7% ASA	3	500.00 ± 25.00	5.0	b

**Table 14:** ANOVA results for Dynamic Creep Modulus of the Modified and Base Asphalt Mixtures.

Condition	F-Value	*p*-Value
Unaged	60.353	<0.001
STA	57.053	<0.001
LTA	45.108	<0.001

**Table 15:** Aging Index of base and modified asphalt mixtures.

Samples	Aging Index (%) STA	Aging Index (%) LTA
Base asphalt mixtures	1.29	2.65
3% ASA	1.25	2.10
5% ASA	1.20	2.00
7% ASA	1.21	3.89

**Table 16:** Statistical summary for Dynamic stability of the Modified and Base Asphalt Mixtures.

Condition	ASA Content	*n*	Mean ± SD	CV (%)	Tukey Letter
Overall	0%	3	282.00 ± 16.92	6.0	c
Overall	3% ASA	3	1105.00 ± 66.30	6.0	b
Overall	5% ASA	3	3791.00 ± 227.46	6.0	a
Overall	7% ASA	3	1212.00 ± 72.72	6.0	b

**Table 17:** ANOVA results for Dynamic stability of the Modified and Base Asphalt Mixtures.

Condition	F-Value	*p*-Value
Overall	449.415	<0.001

**Table 18:** Statistical summary for Moisture Susceptibility of the Modified and Base Asphalt Mixtures.

Condition	ASA Content	*n*	Mean ± SD	CV (%)	Tukey Letter
Overall	0%	3	85.00 ± 0.65	0.8	c
Overall	3% ASA	3	89.00 ± 0.65	0.7	b
Overall	5% ASA	3	94.00 ± 0.65	0.7	a
Overall	7% ASA	3	93.00 ± 0.65	0.7	a

**Table 19:** ANOVA results for Moisture Susceptibility of the Modified and Base Asphalt Mixtures.

Condition	F-Value	*p*-Value
Overall	120.118	<0.001

## Data Availability

The original contributions presented in this study are included in the article. Further inquiries can be directed to the corresponding author.

## Abbreviations

The following abbreviations are used in this manuscript:

ASA	Acrylate Styrene Acrylonitrile
LDPE	Low-density Polyethylene
EVA	Ethyl Vinyl Acetate
PET	Polyethylene Terephthalate
wt.%	Weight of Asphalt Binder
ESALs	Equivalent single axle loads
OBC	Optimum Binder Content
TSR	Tensile Strength Ratio
ITS	Indirect Tensile Strength
FE-SEM	Field Emission Scanning Electron Microscopy
STA	Short-term aging
LTA	Long-term aging
